# *In vitro* activity of cefepime/zidebactam and cefepime/taniborbactam against aztreonam/avibactam-resistant NDM-like-producing *Escherichia coli* clinical isolates

**DOI:** 10.1093/jac/dkad061

**Published:** 2023-03-15

**Authors:** Christophe Le Terrier, Patrice Nordmann, Mustafa Sadek, Laurent Poirel

**Affiliations:** Faculty of Science and Medicine, Emerging Antibiotic Resistance Unit, Medical and Molecular Microbiology, University of Fribourg, Fribourg, Switzerland; Division of Intensive Care Unit, University Hospitals of Geneva, Geneva, Switzerland; Faculty of Science and Medicine, Emerging Antibiotic Resistance Unit, Medical and Molecular Microbiology, University of Fribourg, Fribourg, Switzerland; Swiss National Reference Center for Emerging Antibiotic Resistance, Microbiology Unit, Fribourg, Switzerland; Institute for Microbiology, University of Lausanne and University Hospital Center, Lausanne, Switzerland; Faculty of Science and Medicine, Emerging Antibiotic Resistance Unit, Medical and Molecular Microbiology, University of Fribourg, Fribourg, Switzerland; Department of Food Hygiene and Control, Faculty of Veterinary Medicine, South Valley University, Qena, Egypt; Faculty of Science and Medicine, Emerging Antibiotic Resistance Unit, Medical and Molecular Microbiology, University of Fribourg, Fribourg, Switzerland; Swiss National Reference Center for Emerging Antibiotic Resistance, Microbiology Unit, Fribourg, Switzerland

## Abstract

**Background:**

Aztreonam/avibactam is one of the last therapeutic options for treating infections caused by NDM-like-producing Enterobacterales. However, PBP3-modified and NDM-producing *Escherichia coli* strains that co-produce CMY-42 have been shown to be resistant to this drug combination. The aim of our study was to assess the *in vitro* activity of cefepime/taniborbactam and cefepime/zidebactam against such aztreonam/avibactam-resistant *E. coli* strains.

**Methods:**

MIC values of aztreonam, aztreonam/avibactam, cefepime, cefepime/taniborbactam, cefepime/zidebactam and zidebactam alone were determined for 28 clinical aztreonam/avibactam-resistant *E. coli* isolates. Those isolates produced either NDM-5 (*n* = 24), NDM-4 (*n* = 2) or NDM-1 (*n* = 2), and they all co-produced CMY-42 (*n* = 28). They all harboured a four amino acid insertion in PBP-3 (Tyr-Arg-Ile-Asn or Tyr-Arg-Ile-Lys).

**Results:**

All strains were resistant to aztreonam/avibactam and cefepime, as expected. The resistance rate to cefepime/taniborbactam was 100%, with MIC_50_ and MIC_90_ being at 16 mg/L and 64 mg/L, respectively. Conversely, all strains were susceptible to cefepime/zidebactam, with both MIC_50_ and MIC_90_ at 0.25 mg/L. Notably, all strains showed low MICs for zidebactam alone, with MIC_50_ and MIC_90_ at 0.5 mg/L and 1 mg/L.

**Conclusions:**

Our data highlighted the excellent *in vitro* performance of the newly developed β-lactam/β-lactamase inhibitor combination cefepime/zidebactam against aztreonam/avibactam-resistant *E. coli* strains, suggesting that this combination could be considered as an efficient therapeutic option in this context. Our study also highlights the cross-resistance between acquired resistance to aztreonam/avibactam and the cefepime/taniborbactam combination.

## Introduction

Among the emerging antibiotic resistance mechanisms, the dissemination of enzymatic mechanisms such as MBLs in Enterobacterales is one of the main sources of concern.^1^ Among MBLs, the New Delhi MBL, NDM, is the most commonly reported among *Escherichia coli* isolates, and is often associated with nosocomial infections, leaving very few therapeutic options available.^2^ NDM enzymes confer resistance to all β-lactams including carbapenems, but do not hydrolyse aztreonam.^3^ However, clinical isolates producing MBLs very often co-produce other β-lactamases such as AmpC-type enzymes or ESBLs, compromising the use of aztreonam alone.^4^ The development of new inhibitors, such as avibactam, has enabled new β-lactam/β-lactam inhibitor (BL/BLI) combinations to be developed. Avibactam, which is a diazabicyclooctane (DBO) β-lactamase inhibitor, has the ability to inhibit many of the most common cephalosporinases and ESBLs, but not MBLs.^5,6^ Therefore, the commercial development of the new combination aztreonam/avibactam offers an interesting therapeutic option against MBL-producing Gram-negatives including NDM producers.^7,8^ Many *in vitro* studies identified this combination as one of the most effective against MBL-producing Enterobacterales. Hence, in a recent systematic review combining *in vitro* and *in vivo* studies, Mauri et *al.*^8^ reported a high antimicrobial activity of aztreonam/avibactam in 80% of MBL-producing Enterobacterales, particularly against NDM-like producers.

Nevertheless, recent studies identified NDM-like-producing *E. coli* isolates showing reduced susceptibility or resistance to aztreonam/avibactam.^9,10^ This non-susceptibility pattern is explained by modifications of the sequence of the PBP3 protein through specific amino acid insertions (Tyr-Arg-Ile-Asn or Tyr-Arg-Ile-Lys), in association with production of CMY-type AmpC-type β-lactamases (and particularly CMY-42) that possess significant hydrolytic activity against aztreonam.^9^ Considering the worldwide spread of such aztreonam/avibactam-resistant and NDM-like-producing *E. coli*, evaluation of other BL/BLI combinations has therefore to be considered.^11^

The recent development of novel BL/BLI combinations including cefepime/zidebactam (WCK 5107) and cefepime/taniborbactam (VNRX-5133) provides promising alternatives against MBL producers. Indeed, zidebactam belongs to the DBO family and possesses a dual effect of direct antibacterial activity and an ‘enhancer’ effect on the PBP2 while its β-lactam partner (i.e. cefepime or aztreonam) acts on PBP3. On the other hand, taniborbactam is a boronic acid derivative with an ability to inhibit MBLs, including NDM-like (except NDM-9) and VIM-like enzymes.^12^ Both BL/BLI combinations have been tested in several *in vitro* studies and are now undergoing Phase 2 or 3 clinical evaluations.^13–15^

In a recent study, Vázquez-Ucha *et al.* highlighted the effective activities of cefepime/zidebactam and cefepime/taniborbactam on 400 strains of carbapenemase-producing Enterobacterales. Cefepime/zidebactam and cefepime/taniborbactam displayed MICs ≤2 mg/L against 96.4% and 75%, respectively, of the tested MBL-producing clinical isolates.^15^

Because these very latest combinations of cefepime/zidebactam and cefepime/taniborbactam exhibited efficient activity against NDM-like producers (although acting by different mechanisms of action), and may soon be commercially available, the objective of our study was to assess the *in vitro* activity of those two combinations against aztreonam/avibactam-resistant *E. coli* strains resulting from PBP3-modified NDM-like and CMY-42 co-production.

## Materials and methods

To achieve this objective, a total of 28 aztreonam/avibactam-resistant NDM-like-producing *E. coli* clinical isolates were included in this study. The strains originated from Angola (*n* = 9), Switzerland (*n* = 9), Pakistan (*n* = 6), France (*n* = 3) and Cameroon (*n* = 1). Those isolates produced either NDM-5 (*n* = 24), NDM-4 (*n* = 2) or NDM-1 (*n* = 2), and they all co-produced CMY-42 (*n* = 28). They all harboured a four amino acid insertion into the PBP3 protein after residue 333, either Tyr-Arg-Ile-Asn (n = 22) or Tyr-Arg-Ile-Lys (*n* = 6) or YRIK (*n* = 6), as previously published.^9^

MICs were determined by using broth microdilution for aztreonam, aztreonam/avibactam, cefepime, cefepime/zidebactam, cefepime/taniborbactam and zidebactam alone. Cefepime and aztreonam were purchased from Sigma-Aldrich (St Louis, MO, USA), and zidebactam (HY-120859) and taniborbactam (HY-109124) from MedChem Express (Luzern, Switzerland). The concentrations of the zidebactam, taniborbactam and avibactam β-lactamase inhibitors were all fixed at 4 mg/L.^5,15^ MICs were determined in triplicate using broth microdilution in Mueller–Hinton broth (Bio-Rad, Marnes-la-Coquette, France) for all antibiotics or antibiotic combinations listed above. Results were interpreted according to the latest EUCAST breakpoints, and susceptibility breakpoints for the novel BL/BLI combinations were defined by referring to the corresponding β-lactam breakpoints.^16^ MIC_50_ and MIC_90_ values were calculated for reporting results. The reference strain *E. coli* ATCC 25922 was used as quality control for all testing.

In addition to the *E. coli* clinical isolates, and in order to evaluate the impact of the different genetic features already proven to interfere with susceptibility to aztreonam/avibactam, the susceptibility to the novel BL/BLI combinations was determined using a series of *E. coli* MG1655 recombinant strains exhibiting either the Tyr-Arg-Ile-Asn or Tyr-Arg-Ile-Lys amino acid insertions into their PBP3 sequence.^17^ The *bla*_CMY-42_ gene was amplified by PCR using primers CMY-For (5′-AACACACTGATTGCGTCTGACG-3′) and CMY-Rev (5′-GGCAAAATGCGCATGGGATT-3′), and cloned into plasmid pTOPO (Invitrogen), and then the recombinant plasmids were electrotransformed into each of the three *E. coli* MG1655 backgrounds (WT, with Tyr-Arg-Ile-Asn insertion or with Tyr-Arg-Ile-Lys insertion).

## Results and discussion

MIC values obtained for clinical isolates and recombinant *E. coli* strains are listed in Table 1. As expected, all clinical isolates were resistant to aztreonam, aztreonam/avibactam and cefepime. The respective MIC_50_ values were at 32, 8 and 256 mg/L, and MIC_90_ values at 64, 16 and 256 mg/L. Interestingly, all aztreonam/avibactam-resistant clinical isolates tested here showed cross-resistance to cefepime/taniborbactam, with MIC_50_ and MIC_90_ being at 16 mg/L and 64 mg/L, respectively. By contrast, cefepime/zidebactam and zidebactam alone remained very effective, with corresponding MICs still being very low (Table 1). The PBP3 modifications in those different recombinant strains did not impact the MICs for cefepime/zidebactam and zidebactam, highlighting the direct antibacterial activity of the latter, and confirming its enhancing effect on the *E. coli* PBP2. On the other hand, the four amino acid insertion into the PBP3 sequences significantly impacted the MICs of aztreonam, aztreonam/avibactam, cefepime and cefepime/taniborbactam, although not sufficiently to reach resistance breakpoints. Among the different clinical isolates tested, only those exhibiting combined resistance mechanisms including modification of PBP3 and co-production of NDM-like and CMY-42 enzymes were shown to be sufficient to confer resistance to cefepime/taniborbactam.

**Table 1.** Susceptibility testing of clinical *E. coli* isolates and recombinant *E. coli* strains for the different BL/BLI combinations tested

Strain						MIC (µg/mL)^a^					
	ST^b^	Origin of isolation^b^	MBL^c^	Other β-lactamase(s)^c^	PBP3 insertion sequence^d^	ATM	AZA	FEP	FEP-ZID	FEP-TAN	ZID
R27922	—	—	—	—	—	≤0.125	≤0.125	≤0.125	≤0.125	≤0.125	0.25
MG1655	—	—	—	—	—	≤0.125	≤0.125	≤0.125	≤0.125	≤0.125	0.25
MG1655	—	—	—	—	YRIK	**1**	**1**	**0.5**	≤0.125	**0.5**	0.25
MG1655	—	—	—	—	YRIN	**1**	**1**	**0.5**	≤0.125	**0.5**	0.25
MG1655	—	—	—	CMY-42	—	**32**	≤0.125	**0.5**	≤0.125	≤0.125	0.25
MG1655	—	—	—	CMY-42	YRIK	**32**	**2**	**2**	≤0.125	**0.5**	0.25
MG1655	—	—	—	CMY-42	YRIN	**32**	**2**	**2**	≤0.125	**0.5**	0.25
MG1655	—	—	NDM-5	—	—	≤0.125	≤0.125	**8**	≤0.125	**0.5**	0.25
MG1655	—	—	NDM-5	—	YRIK	**0.5**	**0.5**	**128**	≤0.125	**8**	0.25
MG1655	—	—	NDM-5	—	YRIN	**0.5**	**0.5**	**128**	≤0.125	**16**	0.25
R-461	167	France	NDM-1	CMY-42	YRIN	32	16	256	≤0.125	32	0.5
R-3038	10	Angola	NDM-5	CMY-42	YRIN	32	8	256	≤0.125	16	0.5
R-3031	10	Angola	NDM-5	CMY-42	YRIN	128	8	256	≤0.125	16	0.25
N-185	361	Switzerland	NDM-5	CMY-42	YRIN	32	8	256	≤0.125	32	0.5
N-590	167	Switzerland	NDM-5	CMY-42	YRIN	64	8	256	≤0.125	16	0.5
R-460	648	France	NDM-1	CMY-42	YRIN	>256	8	>256	≤0.125	32	0.5
R-3033	10	Angola	NDM-5	CMY-42	YRIN	64	8	256	≤0.125	16	0.5
R-3040	10	Angola	NDM-5	CMY-42	YRIN	64	8	256	≤0.125	16	0.5
R-3043	10	Angola	NDM-5	CMY-42	YRIN	64	8	256	≤0.125	16	0.5
R-3029	10	Angola	NDM-5	CMY-42, CTX-M-group 1	YRIN	32	8	>256	≤0.125	16	0.5
R-3039	10	Angola	NDM-5	CMY-42	YRIN	16	8	256	≤0.125	16	0.5
R-3048	10	Angola	NDM-5	CMY-42	YRIN	16	8	>256	≤0.125	16	0.5
R-3054	10	Angola	NDM-5	CMY-42	YRIN	16	8	>256	≤0.125	16	0.25
N-57	405	Switzerland	NDM-5	CMY-42	YRIK	32	8	>256	≤0.125	64	1
R-466	405	Cameroon	NDM-4	CMY-42, CTX-M-15, OXA-1	YRIK	>256	8	>256	≤0.125	32	1
R-2222	9747	France	NDM-4	CMY-42	YRIK	>256	8	>256	≤0.125	16	0.25
148C	167	Pakistan	NDM-5	CMY-42, TEM-1B	YRIN	32	8	>256	≤0.125	64	0.25
272A	167	Pakistan	NDM-5	CMY-42, TEM-1B	YRIN	64	8	>256	≤0.125	64	0.25
278A	167	Pakistan	NDM-5	CMY-42, TEM-1B	YRIN	32	8	>256	≤0.125	32	0.25
N1153	167	Switzerland	NDM-5	CMY-2, CTX-M-15, OXA-1, TEM-1B	YRIK	>256	8	>256	≤0.125	64	0.125
N1146	167	Switzerland	NDM-5	CMY-42, TEM-1B-like	YRIN	32	8	128	≤0.125	16	0.125
240F	205	Pakistan	NDM-5	CMY-42, TEM-1B	YRIK	32	16	256	≤0.125	16	1
N1013	361	Switzerland	NDM-5	CMY-42	YRIN	128	8	256	≤0.125	64	0.25
N1416	405	Switzerland	NDM-5	CMY-42, CTX-M-15, OXA-1, TEM-1B	YRIK	>256	16	>256	≤0.125	64	1
142A	61	Pakistan	NDM-5	CMY-42, TEM-1B	YRIN	64	8	256	≤0.125	32	0.5
N1470	617	Switzerland	NDM-5	CMY-42	YRIN	64	16	>256	≤0.125	16	0.25
N1076	940	Switzerland	NDM-5	CMY-42, TEM-1B	YRIN	64	8	>256	≤0.125	64	1
246A	2659	Pakistan	NDM-5	CMY-131, TEM-1B	YRIN	64	8	>256	≤0.125	8	0.125

Bold MIC values correspond to a significantly elevated MIC value in the recombinant *E. coli* strains compared with WT *E. coli* MG1655. ATM, aztreonam; AZA, aztreonam/avibactam; FEP, cefepime; FEP-TAN, cefepime/taniborbactam; FEP-ZID, cefepime/zidebactam; ZID, zidebactam. ZID and TAN were used at a fixed concentration of 4 µg/mL.

Dash indicates not applicable. ST, sequence type.

Dash indicates no β-lactamase.

Dash indicates no insertion of amino acids in the PBP3 sequence.

### Conclusion

This study highlighted that the efficacy of the novel BL/BLI combination cefepime/taniborbactam, developed to be efficient against NDM-producing isolates, was significantly impacted by the same resistance mechanisms that have been shown to counteract the efficacy of aztreonam/avibactam, namely the co-production of CMY-42 and NDM and the modification of the PBP3 target. On the other hand, our data showed that cefepime/zidebactam was an excellent therapeutic option against those MBL producers, mainly related to the antibacterial and enhancing activity of zidebactam. This combination therefore constitutes one of the ultimate treatment options in such contexts.
